# Heart Failure in the Modern Era: A Narrative Overview of Recent Research from 2022–2025

**DOI:** 10.3390/jcdd12120484

**Published:** 2025-12-10

**Authors:** Michał Wilk, Rafał Tymków

**Affiliations:** 1 Student Scientific Organization, Institute of Heart Diseases, Wroclaw Medical University, 50-556 Wrocław, Poland; 2 Institute of Heart Diseases, Wroclaw Medical University, 50-556 Wrocław, Poland

**Keywords:** heart failure, cardiomyopathies, amyloidosis, heart failure therapies

## Abstract

Heart failure (HF) remains a major challenge in cardiovascular medicine, contributing to high global rates of hospitalization and mortality. Recent research (2022–2025) has emphasized its heterogeneity, highlighting distinct phenotypes—HFpEF, HFmrEF, and HFrEF—driven by mechanisms such as chronic inflammation, myocardial fibrosis, and neurohormonal imbalance. Advances in therapy, particularly with sodium–glucose cotransporter-2 inhibitors (SGLT2i), angiotensin receptor–neprilysin inhibitors (ARNI), and iron supplementation, have reshaped treatment strategies. Moreover, the growing recognition of overlaps between HF and cardiomyopathies such as hypertrophic, Takotsubo, and amyloidosis underscores the need for integrated care. This review summarizes recent findings from leading journals, mapping the evolving understanding of HF pathophysiology and management, and outlining emerging directions for research and clinical practice.

## 1. Introduction

Heart failure (HF) remains one of the most pressing challenges in contemporary cardiovascular medicine, continuing to drive high rates of hospitalization and mortality worldwide. At the same time, it is increasingly recognized as a heterogeneous syndrome, shaped by diverse biological mechanisms and complex clinical trajectories. Over the past two years, a wave of studies published in the most recent issues of the *European Journal of Heart Failure*, *Circulation HF*, *ESC Heart Failure* and *JACC HF* have provided valuable progress across multiple domains. Considerable attention has been directed to the HF phenotypes—heart failure with preserved ejection fraction (HFpEF), heart failure with mildly reduced ejection fraction (HFmrEF), and heart failure with reduced ejection fraction (HFrEF)—with new insights into chronic inflammation, myocardial fibrosis, neurohormonal imbalance, and the role of systemic comorbidities. Alongside these mechanistic observations, advances in therapy are striking: sodium–glucose cotransporter-2 inhibitors (SGLT2i) and angiotensin receptor–neprilysin inhibitors (ARNI) are increasingly regarded as therapeutic pillars, while renewed focus on iron supplementation adds further depth to the treatment landscape. It is also worth noting that HF intersects with other conditions, such as hypertrophic cardiomyopathy (HCM), Takotsubo cardiomyopathy, and cardiac amyloidosis (CA), broadening the scope of investigation and underscoring the need for integrated care strategies. Importantly, obesity, inflammation and fibrosis represent central pathophysiological mechanisms that interconnect HFpEF, HCM, and amyloidosis, driving structural and functional myocardial changes, contributing to clinical deterioration, and highlighting shared targets for potential therapeutic intervention across these distinct HF origins. Notably, only recently has Milton Packer proposed the “adipokine hypothesis,” which positions adipose tissue and adipokines as a key element of HFpEF pathophysiology. By bringing together these most up-to-date contributions, this review seeks to map out the current state of knowledge, highlight ongoing challenges, and outline future directions in the management of HF. We believe that a comprehensive collection of the latest data on HF will enable us to create a more comprehensive and nuanced view of the complexity of the problem and the dynamic nature of our efforts to find answers to many questions related to this subject.

## 2. Materials and Methods

This review was designed as a narrative synthesis rather than a quantitative analysis. We examined work produced from January 2022 to July 2025 and published in the most recent volumes of the *European Journal of Heart Failure*, *ESC Heart Failure*, *Circulation*, and *JACC*, with particular emphasis on their specialist journals: JACC HF and Circulation HF, with a search scope of 2022–2025. Some topics were supplemented by publications from *NEJM*, *The Lancet*, and *Nature*. All journals were reviewed using the same standardized approach. Relevant articles were identified through a systematic screening of the journal’s archive and bibliographic databases (PubMed, Web of Science, and Scopus) using keywords such as “SGLT-2i,” “HFpEF,” “HFrEF,” “cardiomyopathy,” “therapy,” and “inflammation.” Both original investigations and review articles were considered, provided they addressed clinically or mechanistically significant aspects of HF. When several publications addressed a similar topic, preference was given to those offering the most comprehensive, methodologically robust, or clinically relevant evidence. No statistical pooling was attempted; instead, the evidence was organized thematically. Publications were clustered into major areas of focus, including HF phenotypes, pharmacological therapies, and cardiomyopathies. The findings were then qualitatively integrated to capture key developments, highlight persistent uncertainties, and emphasize their implications for clinical practice.

## 3. Three Faces of Heart Failure: HFpEF, HFmrEF, and HFrEF

### 3.1. Heart Failure with Preserved Ejection Fraction

HFpEF is a heart failure (HF) subtype of diverse etiology that is responsible for more than half of all HF hospitalizations [1]. The complexity of this type of heart failure results from the variety of pathophysiological factors underlying the development of the disease and the interactions between these factors. For this reason, our understanding of HFpEF treatment, course, and risk stratification is insufficient; this has led to considerable interest in this subject, as evidenced by the many studies published over the past two years [1,2,3,4,5,6,7,8,9,10,11,12,13,14,15,16,17,18,19,20,21,22,23,24,25,26,27,28,29,30,31,32,33,34,35,36,37,38,39,40,41,42,43,44,45,46,47,48,49,50,51,52,53,54,55,56,57,58,59,60,61,62,63,64,65,66,67,68,69,70,71,72,73,74,75,76,77,78,79,80,81,82,83,84,85,86,87,88,89,90]. Only recently, a new hypothesis for HFpEF development and progression has been proposed, with metabolic dysfunction (adipokine dysregulation) as the main trigger in this process. The primary interest in the pathophysiological context relates to processes such as chronic low-grade inflammation [4,6,13,17,37,42,90], myocardial fibrosis [7,34,42,90], neurohormonal activation [10,41,50], and oxidative stress [13,43,68]. Additionally, numerous authors draw attention to the contribution of comorbidities to the development of HFpEF [3,10,13,16,19,40,46,47,51,55,62,68,74,75,87], most importantly, obesity [2,3,10,11,13,25,26,50,68,71,72,75], with particular emphasis on epicardial adipose tissue (EAT) [11,20,91], and secondly, type 2 diabetes mellitus (T2DM) [2,3,13,14,15,18,19,50,51,53,68,72,74,87]. Despite the etiology of HFpEF remaining unclear, perpetuating inflammation caused by excess adipokines, as well as microvascular damage resulting from oxidative stress, appears to play a significant role [2,10]. This is consistent with the theory of its influence on HFpEF. Moreover, only recently, Milton Packer has proposed the adipokine-centric theory of HFpEF development and progression [80]. This theory moves away from viewing HFpEF as a condition driven primarily by comorbidities and instead reframes it as a disease rooted in adipose-tissue abnormalities that are independent of accompanying comorbidities. Adipose-tissue dysfunction leads not only to disrupted production of specific adipokine domains but also to disturbed relationships and balance between these domains [80]. Thus, we strongly believe this theory will be further developed in future studies. Moreover, some authors draw attention to the fact that smoldering inflammation, in the long run, also leads to cardiac and vascular fibrosis [7,34,42], consequent stiffness of the heart and arteries [3,42,60,82], diastolic dysfunction [3,6,12,42,43,44,50], and, ultimately, hemodynamic consequences [26,36,43,64,69,71].

Among the comorbidities associated with HFpEF, atrial fibrillation (AF) seems to play a very important role [8,19,61,74,81]. A study by Chieng et al. report a 51% prevalence of AF in HF and a 21% prevalence of HFpEF in patients with AF, while noting that HFpEF is most likely underdiagnosed in patients with AF due to common symptomatology [61]. The method of treating AF in patients with HF still leaves many questions, but one possible approach is catheter-directed ablation [61,81]. Proponents of the aforementioned method point to the fact that restoring sinus rhythm with this procedure in a selected subgroup of patients with HFpEF and concomitant AF resulted in improvement in parameters such as peak exercise PCWP (30.4 ± 4.2 to 25.4 ± 4.5 mm Hg; *p* < 0.01), peak relative VO2 (20.2 ± 5.9 to 23.1 ± 7.2 mL/kg/min; *p* < 0.01), and a decrease in N-terminal pro–B-type natriuretic peptide levels (794 ± 698 to 141 ± 60 ng/L; *p* = 0.04) [61]. The potential mechanism responsible for the improvement in parameters is considered to be normalized atrioventricular hemodynamics, alleviated atrial overload, and, consequently, reduced pressure in the pulmonary veins [61]. Researchers also show a correlation between an improvement in symptoms severity, exercise capacity, and overall quality of life (QoL), as measured by the Minnesota Living with Heart Failure questionnaire [61].

From a diagnostic perspective, HFpEF remains a challenge because it may remain latent for a long period without producing apparent symptoms, which is why some authors focus their efforts on finding the ideal biomarker that would facilitate diagnosis [2,21,28,47,48,53,68]. In the classic approach to biomarkers, a correlation between the occurrence of HFpEF and congestion has been demonstrated for carbohydrate antigen 125 (CA-125) [34], as well as for high-sensitivity C-reactive protein (hsCRP) [4]. In addition to the standard markers, the roles of exercise tests and imaging studies appear to be becoming increasingly significant [3,10,26,37,55]. Exercise intolerance, demonstrated by a vast proportion of HFpEF patients, may be one of the earliest detectable indicators of the disease, as some researchers have pointed out [3,10,11,26,36,47]. It is worth noting that “simple” exercise tests, like cycloergometry, seem to provide important data on patients with HFpEF [3,26,36,43,47,65,83]. For instance, a study by Nan Tie et al. found an association between hyperlactatemia and a decrease in VO2 max in the HFpEF subset compared to healthy volunteers, which could be considered an additional diagnostic measure [36].

Interestingly, the diagnosis of HFpEF can be supported by modern imaging techniques, which add diagnostic value to standardized procedures such as echocardiography. Accordingly, a study using a method called pericoronary fat attenuation on coronary computed tomography angiography (PCATA) was performed. PCATA allows the detection of pericoronary adipose tissue, which may be a source of cytokines mediating chronic inflammation (adipokines), that induce a molecular cascade leading to HFpEF [37]. The authors state that high PCATA was associated with the presence of HFpEF, supporting the immune-derived background [37]. Although PCATA cannot directly detect cytokine production; however, it can identify coronary segments that exhibit imaging features consistent with underlying inflammatory activity [37].

Lastly, the heterogeneity of HFpEF prompted scientists to divide HFpEF into phenotypes to facilitate management and treatment [3,6,10,14,25,32,42,47,50,51,62,70]. This process might be improved by implementing machine learning, as demonstrated in a Japanese population, where this mentioned method allowed the classification of HFpEF patients into three phenotypic groups: atherosclerosis and chronic kidney disease (CKD); atrial fibrillation (AF), and younger groups with left ventricular hypertrophy (LVH) [25]. Thanks to this and the abundance of data regarding HFpEF, machine learning and human-created algorithms enable the improvement of risk stratification among HFpEF patients [15,19,24,89]. Additionally, the study by Wu et al. shows that the population of undiagnosed patients with HFpEF may be a more serious problem than it seemed, which is particularly important because this group of patients is burdened with higher 5-year mortality despite being less comorbid and experiencing fewer acute cardiovascular events [89]. This study demonstrated correlations between the use of AI-based diagnostic models and the better identification of patients with underdiagnosed HFpEF, which could enable this group to benefit more effectively from specialist cardiology care [89].

Recent clinical trials and large-scale analyses evaluating GLP-1 receptor agonists, including semaglutide and tirzepatide, have demonstrated significant clinical benefits in patients with HFpEF, particularly in those with obesity [91,92,93,94,95]. In addition to improving symptoms and exercise tolerance, more signals of health benefits and reduced HF related events have emerged, providing some of the first robust evidence, that therapies directly targeting obesity can modify the clinical course of HFpEF [91,93,95,96,97,98]. The effect of weight loss, lowered inflammatory markers, and favorable changes in adipokine signaling observed with GLP-1 agonists further support Packer’s concept [80,91,93,96,99]. Collectively, these data indicate a potential cardioprotective effect of GLP-1-based therapies that may be mediated by modulation of dysfunctional adipose tissue, which is consistent with the approach proposed in the adipokine hypothesis.

In summary, HFpEF results from a complex interplay of inflammatory, metabolic, and hemodynamic pathways. Chronic low-grade inflammation and adipokine disbalance, often triggered by obesity and diabetes (metabolic factor), contribute to myocardial fibrosis and vascular stiffness, leading to diastolic dysfunction and hemodynamic alterations. Metabolic dysregulation, EAT activity, and neurohormonal imbalances further exacerbate these processes, while AF and other comorbidities modulate the clinical presentation and prognosis. Taken together, these interrelated mechanisms underscore the heterogeneity of HFpEF, emphasizing the need for phenotype-specific approaches to diagnosis, risk stratification, and therapy, and highlighting persistent knowledge gaps that require further investigation.

### 3.2. Heart Failure with Mildly Reduced Ejection Fraction

HFmrEF is a category of HF that lies between HFpEF and HFrEF. More specifically, it refers to a group of patients whose ejection fraction (EF) lies between 41% and 49%. Because this is a relatively new term—it was first introduced in the 2016 ESC guidelines as a heart failure with a mid-range ejection fraction—it was rather perceived as a gray area among other HF phenotypes. In response to the post-2016 HF classification, a growing number of studies are focusing their efforts on broadening our understanding of HFmrEF, thereby informing therapeutic approaches for this group of patients [100,101,102,103,104,105,106,107,108,109,110,111,112,113,114,115,116,117,118,119,120,121,122,123,124,125,126].

The most important aspect of HFmrEF research seems to be pharmacotherapy and whether guideline-directed medical therapy (GDMT) designed for HFrEF is adequate and equally effective in this group of patients. Additionally, apart from MRA and SGLT-2i, most data for HFmrEF are from post hoc or subsets of analyses from previous HF trials [109]. Therefore, we highlight a great need for studies confirming the effectiveness of specific drug groups, especially in relation to the four-pillar concept of HFrEF treatment. Studies on the use of beta blockers in patients with HFmrEF report that they do not appear to be associated with a worse prognosis [102,112,114]. However, it is worth noting analyses show that as EFs rise above 40%, there is a correlation with a decrease in the drug’s benefits decrease [102]. Importantly, a possible benefit was also observed in the HFmrEF group, but among patients with an EF around 60%, the risk of hospitalization significantly increased, while the survivability decreased [102].

Another important group of drugs whose effectiveness is being verified for treatment of HFmrEF is SGLT2i [100,104,107,117,125]. The SGLT2i are the first class of drugs effective in the whole spectrum of EF in HF. The results of SGLT2i studies are consistent with the previous results of research on their effectiveness in HFrEF, indicating precisely their multifaceted beneficial effect on the course of HF [101,103,120,126]. The most important correlations include the beneficial effect of empagliflozin on anti-inflammatory and antioxidant effects of this drug [107], and an overall improvement in QoL after dapagliflozin implementation, expressed in indicators such as the Q-5D VAS and EQ-5D index, regardless of EF, which shows that the effect of dapagliflozin goes beyond the alleviation of HF symptoms alone [125]. Another study also shows that the benefits of dapagliflozin persisted during the pandemic, regardless of COVID-19 cases, confirming the drug’s effectiveness and the stability of therapy even in difficult clinical conditions [104]. It is also worth mentioning the association between the use of dapagliflozin and a reduced risk of HF exacerbation regardless of the QRS complex length, which creates an opportunity for complementary therapy for patients insufficiently treated with cardiac resynchronization therapy (CRT) [100]. Therefore, based on available studies, it can be concluded that SGLT2i have a beneficial and multifactorial effect on the treatment of HFmrEF.

Another group of medications being studied in the context of HFmrEF is mineralocorticoid receptor antagonists (MRAs) [106,122]. A FINEARTS-HF study of a new member of this group, finerenone, showed that its use in HFmrEF patients led to a reduced risk of cardiovascular death and hospitalization due to HF and cardiovascular death, as well as improved QoL and reduced HF symptoms [106,121]. It is worth noting that finerenone was safe and well-tolerated in this study, regardless of patient age—the incidence of the primary outcome increased with age, yet finerenone consistently reduced the risk across all age quartiles, with rate ratios of 0.70 (95% CI, 0.53–0.92) in Q1, 0.83 (95% CI, 0.64–1.07) in Q2, 0.98 (95% CI, 0.76–1.26) in Q3, and 0.85 (95% CI, 0.67–1.07) in Q4, highlighting its efficacy even in older, higher-risk patients [106].

Regarding risk factors and survival assessment, an important analysis was performed to assess the impact of gender on prognosis in HFmrEF. A study by Keshvani et al. found an association between female gender and decreased 5-year mortality, similar to that in the HFrEF group [111]. It is worth noting that despite lower mortality in women, the analysis showed that women had a higher risk of rehospitalization than men and lost 2–3 additional years of life due to HF hospitalization, which was due to their longer average life expectancy [111]. Furthermore, women with HFmrEF had a lower burden of ischemic heart disease, atrial fibrillation, and renal disease, which partially explains the lower mortality [111]. This indicates that gender is an important risk modifier in the HFmrEF group, and partially highlights the need to intensify treatment in women in order to avoid hospitalization, which is associated with greater complications.

Despite this, HFmrEF remains a controversial phenotype, with ongoing debate over whether it represents a distinct entity or a transitional stage between HFrEF and HFpEF. Additionally, HFmrEF continues to pose clinical challenges due to its heterogeneous clinical presentation, variable prognosis, and limited evidence regarding optimal treatment.

### 3.3. Heart Failure with Reduced Ejection Fraction

Although HFpEF has received significant scientific attention in recent years, HFrEF remains an interesting area of research, as recent data show [127,128,129,130,131,132,133,134,135,136,137,138,139,140,141,142,143,144,145,146,147,148,149,150,151,152,153,154,155,156,157,158,159,160,161,162,163,164,165,166,167,168,169,170,171,172,173,174,175,176,177]. Despite established treatment based on the four pillars, questions regarding optimal therapy remains. In addition to standard medication, innovative methods using neuromodulation and novel drugs are being tested [129,132,133,138,140,146,152,156,159,162,163,164,165,168,169,170,177]. One of these methods is baroreflex activation therapy (BAT), which counteracts autonomic system disbalance [129,138,152,163,177]. Although BAT was originally designed for patients resistant to standard hypertension (HT) treatment, studies suggest its effectiveness and safety in patients with HFrEF [129,138,163,177]. BAT shows correlation with improved NYHA functional class, QoL, 6-min walking test results, and N-terminal pro-B-type natriuretic peptide (NT-proBNP) levels [129,138,163,177]. Researchers also point out an association with slightly improved left ventricular contractility. In addition, the number of hospitalizations due to HF following device implementation was lower than expected as well [129,138]. It is worth noting that BAT does not appear to impact mortality and rehospitalization rates in patients with advanced HFrEF, defined as NYHA Class IV, NT-proBNP levels > 1600 pg/mL, or estimated glomerular filtration rate (eGFR) < 30 mL/min; therefore, the subset that could benefit from the use of BAT remains undefined [129,138]. Although BAT did not demonstrate a significant difference in primary endpoints compared with the control group, which were CV mortality and HF morbidity, long-term results indicated that BAT provided a safe, effective, and sustained improvement in the functional status of patients with HFrEF [163,177].

In the context of new drugs that could be included in standard HFrEF pharmacotherapy, vericiguat, already called by some the fifth pillar of HF therapy, comes to the fore [132,133,146,156,159,162,164,165,168,169,170,178]. Insights into the VICTORIA trial and analyses of national HF registries, (for instance, the Swedish Heart Failure Registry) have shown that vericiguat added to standard HFrEF treatment significantly reduces the risk of the composite endpoint of CV death and hospitalization due to HF [133,156,164,178]. Additionally, a study by Suzuki et al. shows a correlation between improvement of hemodynamic parameters, such as normalization of pulmonary artery wedge pressure (PAWP), and the use of vericiguat in patients with HFrEF [146]. Due to the unique mechanism of action, the issue of safety and appropriate selection is still being investigated; however, available data suggest that vericiguat is safe among patients meeting the inclusion criteria for the VICTORIA study and can be successfully implemented in a HFrEF GDMT, regardless of adherence to primary therapy and diabetes status [132,133,162,166,178]. We are awaiting the results of the VICTOR trial, and planned meta-analysis of both trials. While the four established pillars of HFrEF remain the cornerstone of therapy, vericiguat has been shown to provide additional risk reduction in CV death and hospitalization for HFrEF, offering additional benefits when combined with standard treatment.

In the last two years, studies have appeared showing correlations between HFrEF and diseases such as depression, obstructive sleep apnea (OSA), and bone diseases and HFrEF [137,143,148]. In a study on OSA, patients with coexisting OSA and HFrEF had a 23.4% higher risk of rehospitalization, with 48.9% of these rehospitalizations directly related to HFrEF and occurring within 30 days of the initial hospitalization [137]. Patients with HFrEF and depressive symptoms showed an association between increased risk of hospitalization or death due to HF, compared to patients with HFrEF without depression (HR: 1.37) [143]. Study results were also associated with unfavorable increases in BNP and decreases in EF [143]. Lastly, in a study of bone diseases such as osteoporosis or vertebral fractures (VF), HFrEF affected one in four patients. The authors note that patients with concomitant VF and HFrEF had twice the risk of CV death or hospitalization due to worsening heart failure (WHF), which is a clinically significant deterioration in HF symptoms or need for therapy escalation [148]. According to the authors, the co-occurrence of these diseases is clinically relevant. A summary of the most important findings by HF phenotype is presented in Table S1.

## 4. Advancing Heart Failure Care

### 4.1. Glucagon-like Peptide-1 Receptor Agonists

Glucagon-like peptide-1 receptor agonists (GLP-1 RAs) have gained increasing attention as therapeutic agents with relevance to cardiometabolic disease and HF. Their effects extend beyond glycemic control and weight reduction, influencing hemodynamics, cardiac structure, and, potentially, cardiomyocyte function. These properties appear particularly relevant in obesity-related HFpEF, although available data indicate applicability across a broader range of HF phenotypes.

In studies employing invasive hemodynamic monitoring, treatment with semaglutide or tirzepatide over six months was associated with measurable reductions in systolic, diastolic, and mean pulmonary artery pressures. These changes occurred despite stable HF GDMT and without adjustments in diuretic dosing, and they correlated with the degree of weight loss [179]. Structural adaptations have also been documented. In the SUMMIT CMR substudy, tirzepatide reduced left ventricular mass and paracardiac adipose tissue volume relative to placebo, accompanied by shifts in cardiac chamber volumes. These findings align with the known influence of adiposity and metabolic load on cardiac structure in obesity-related HFpEF [90].

Experimental work in isolated human ventricular myocardium provides additional mechanistic context. Semaglutide reduced late sodium current, decreased sarcoplasmic reticulum calcium leak, and improved systolic calcium transients and contractile performance in both HFpEF-like and HFrEF myocardial samples. These effects occurred through GLP-1 receptor–dependent pathways and were comparable to CaMKII inhibition, thereby offering a mechanistic explanation for some of the clinical observations [180].

Clinical outcome data further support the relevance of this drug class in HF. In the SELECT trial, semaglutide (2.4 mg) reduced major adverse cardiovascular events and a composite of CV death or HF events in patients with established atherosclerotic cardiovascular disease (CVD) and who were overweight or obese, including individuals with pre-existing HF. The effect was consistent across HFpEF and HFrEF subtypes, and no safety concerns specific to reduced ejection fraction were observed [96]. In parallel, dedicated HFpEF studies—STEP-HFpEF and STEP-HFpEF DM—demonstrated improvements in symptoms, physical limitations, functional capacity, inflammation markers, and body weight with semaglutide therapy, regardless of the presence of T2DM [92,93].

Evidence from meta-analyses indicates that GLP-1 RAs consistently reduce rates of major cardiovascular events, HF hospitalizations, and kidney disease progression, with treatment effects preserved irrespective of concurrent SGLT2 inhibitor use. This suggests complementary rather than overlapping mechanisms [99]. Modeling analyses also propose that integrating GLP-1 RAs with other modern cardiometabolic therapies, including SGLT2i and MRAs, may enhance long-term event-free survival [181].

Overall, the available evidence indicates that GLP-1 receptor agonists modify several processes relevant to HF pathophysiology, including metabolic loading conditions, adipose tissue distribution, and cellular ion handling. Their strongest evidence base currently pertains to patients with obesity-related HFpEF, though emerging data suggest broader applicability. Ongoing trials are expected to refine their positioning within comprehensive HF management strategies.

### 4.2. Sodium-Glucose Cotransporter 2 Inhibitors

SGLT2i have proven to be a key element in the modern HF therapy, regardless of the classification based on EF, hence the great interest in them [178,182,183,184,185,186,187,188,189,190,191,192,193,194,195,196,197,198,199,200,201,202,203,204,205,206,207,208,209,210,211,212,213,214,215,216,217,218,219,220,221,222,223,224,225,226,227,228,229,230,231,232]. Currently, researchers are trying to determine the particular components of the multifaceted beneficial effects of these drugs [182,183,184,186,187,191,192,195,224,225,228,232].

Much of the current research regarding SGLT-2i concerns interactions with kidney function, especially in the context of co-occurring HF, which is why we repeatedly see references to cardiorenal interactions [184,190,195,208,209,223,226]. Some researchers even suggest that the main component of the beneficial SGLT-2i’s beneficial effect in HF may result primarily from preserving renal function and reducing the burden exerted by other drugs; however, it is worth remembering that the exact mechanism is not known [184,189,190,226].

After the EMPEROR-preserved study, the view on the appropriateness of SGLT-2i use in HFpEF seems to be established [189,190,197,198,223,227]. The valuable effects of SGLT-2i in HFpEF are being linked to processes such as anti-inflammatory action,, anti-fibrotic action, fat tissue mobilization, decongestion, and even ferroptosis [182,187,192,195,196,197,202,203,204,224,232]. Moreover, a study by Clemmer et al. supports the use of SGLT-2i in HFpEF, mainly because of the positive effects that these drugs exert on HFpEF comorbidities, such as CKD and HT [189]. Additionally, researchers confirm the efficacy of empagliflozin in reducing the composite endpoint—hospitalization due to HF and CV death, regardless of EF; presence/absence of AF; CKD; obesity; MRA use; and differences in eGFR or liver parameters—confirming its strong position in HF therapy [205,206,209,210,211,212,214,215,223,224,226,227]. Regarding the beneficial effect of SGLT2i on kidney function and HFpEF comorbidities, in this case obesity, it is worth paying attention to the results of the EMPEROR-PRESERVED study analyses, such as the slowing down the decline of GFR (1.31–1.43 mL/min/1.73 m^2^/year) and the significant effect on weight loss, depending on BMI, in the 52nd week of observation (from –0.6 kg [BMI < 25] to –2.7 kg [BMI ≥ 40], *p*-trend = 0.016) [223,226]. That is why empagliflozin and its multidirectional modality are considered effective and cost-efficient worldwide, as the authors point out; in many cases; it allows several problems to be addressed at once [192,197,198].

While empagliflozin and dapagliflozin remain in the spotlight, a few studies have emerged on other SGLT2i drugs, such as canagliflozin and sotagliflozin [221,229,230]. A study by Pitt et al. on the efficacy of sotagliflozin in reducing HF readmissions showed a positive association between its use before hospital discharge in patients hospitalized for WHF with concomitant T2DM, and a significant reduction in CV deaths and heart failure-related events within 30 and 90 days after discharge: the primary endpoint at 90 days after discharge (HR: 0.54 [95% CI: 0.35–0.82]; *p* = 0.004), at 30 days (HR: 0.49 [95% CI: 0.27–0.91]; *p* = 0.023), and all-cause mortality at 90 days (HR: 0.39 [95% CI: 0.17–0.88]; *p* = 0.024) [221]. A similar effect on reducing HF hospitalizations was observed in a study on canagliflozin, with particular emphasis on the beneficial effects of this SGLT2i across the entire spectrum of renal function, defined by eGFR; canagliflozin significantly reduced the number of CV deaths and the total number of HF hospitalizations (mean event rate 0.72, 95% CI 0.65–0.80) [229]. These studies indicate a positive trend of an increasing number of SGLT2i representatives, confirming their effectiveness in the treatment of HF, which directly translates into the expansion of the list of available drugs. Although the effect of reducing CV mortality and HF hospitalization may seem similar for canagliflozin, sotagliflozin, and empagliflozin, comparing the effectiveness of the new SGLT2i representatives with empagliflozin remains difficult due to very different populations, endpoints, and follow-up times. Finally, interesting and novel directions of research related to SGLT-2i concern aspects such as the effect in the population with advanced HF and the impact on the course of oncological therapy in patients with comorbidities [233,234]. Novo et al. suggest that the use of SGLT-2i in the population actively treated oncologically with HF and T2DM significantly reduces the risk of all-cause mortality and HF hospitalization due to reduced cardiotoxicity; however, they also emphasize the need to confirm these data in RCT [233]. On the other hand, Nuzzi et al. show that the use of SGLT2i in the population with advanced HF did not result in a significant effect on NTproBNP concentration, LVEF value, or NYHA class, while the drugs are well tolerated. This makes it necessary to precisely define the population that clearly benefits from the therapy [234].

### 4.3. Angiotensin Receptor-Neprilysin Inhibitors

Even though ARNI have proven their effectiveness in the PARADIGM-HF study for reducing the risk of death and hospitalization for HFrEF, they still remain an interesting area of investigation regarding various therapeutic aspects [235,236,237,238,239,240,241,242,243,244,245,246,247,248,249,250,251,252,253,254,255,256]. After the PARAGON-HF trial failed to achieve statistical significance in reducing the composite endpoint (the sum of HF hospitalizations and CV deaths) for the population with an EF above 45%, an important question arose as to who exactly might benefit from this drug [252]. Given the favorable results of the PARADIGM-HF trial, a gray area emerged that needed to be explored, and this was addressed by the investigators responsible for the PARAGLIDE-HF trial [251].

As the aforementioned study demonstrated, in patients with a recent episode of WHF and an EF above 40%, better reductions in NT-pro-BNP were observed after ARNI than after valsartan alone [251]. Furthermore, the effect was most pronounced in subgroups (primarily women) and in patients with EF > 40% but <60% [251]. Therefore, despite the positive reception of the reported study, PARAGLIDE-HF also failed to answer the question of whether the entire HFpEF population could benefit from ARNI [251]. Nevertheless, this is an important step in defining the precise group of patients eligible for this drug, and as can be observed, the positive association between ARNI use and improved survival is particularly evident for patients with HFrEF and HFmrEF. Regarding the drug’s safety, it is worth mentioning that although it demonstrated fewer renal function deteriorations in the Sac/Val group (OR 0.61), this was complicated by a higher risk of symptomatic hypotension (OR 1.73) [251].

Despite a good response in two of the described groups of patients suffering from HF, little is known about how other factors influence ARNI therapy; for example, concomitant therapy with SGLT2i may increase the risk of hypotension, as suggested by some researchers, but there is no hard data on this subject [248,256]. Another important question raised by Bozkurt et al. is whether increasing natriuretic peptide levels and inhibiting the action of neprilysin, which breaks down numerous biologically active peptides along with natriuretic peptides (such as bradykinin, adrenomedullin, angiotensin, and endothelin) has a long-term effect on parameters (such as albuminuria, obesity, glycemic and lipid control, blood pressure, and cognitive function), as data on this subject are also lacking, but some clues are starting to emerge on this subject [248].

On the other hand, some studies show correlations between the use of ARNI and the reversal of negative cardiac remodeling, with subsequent improvement in the ventricular dimensions and systolic parameters [239,240]. Accordingly, hemodynamic forces (HDF), defined as intraventricular pressure gradients averaged over the left ventricular volume during the cardiac cycle, also correlated with the response to treatment [237]. Described correlations may be observed thanks to serial NT-proBNP measurements during treatment, which, in turn, seems to be important in assessing the reversal of cardiac remodeling [241].

In the context of noteworthy situations in which ARNI therapy could be beneficial, we point out to patients from PARAGON-HF with EF > 45% who were analyzed for monitoring response to treatment using red cell distribution width (RDW). It was observed that ARNI therapy does not significantly affect the RDW value (−0.09%), and correlates well with major cardiovascular events, including HF hospitalizations and CV deaths, which may provide important information in this group of patients [235]. Also noteworthy is that there is growing evidence that patients exposed to cardiotoxicity during chemotherapy for breast cancer may also benefit from ARNI use [243].

Finally, much is known about ARNI in the context of assessing and improving the prognosis of patients with HFrEF, but the analysis by Mebazaa et al. found a significant correlation between the use of sacubitril/valsartan and better diuretic and natriuretic responses, weight loss, and better adaptation to stressors that potentially cause fluid overload [250]. According to the authors, after initiation of sacubitril/valsartan, patients’ diuresis and natriuresis in response to the fluid overload test with Ringer’s administration significantly increased (mean difference: 38.8 [SD 17.38] mL, *p* = 0.0040 and 9.6 [SD 2.02] mmol, *p* < 0.0001, respectively). Compared to baseline, increased natriuresis was also observed in the sacubitril/valsartan group after furosemide administration (9.8 [SD 5.13] mmol, *p* = 0.0167), which may suggest that ARNI have a beneficial effect in sensitizing patients to the effects of loop diuretics, which is a common problem in patients with HFrEF [250]. Moreover, there is a growing concept that optimizing GDMT, which counterbalances the neurohormonal drive, is associated with more effective and durable decongestion, as well as reduced diuretic requirements [246,247].

### 4.4. Iron Supplementation and Metabolism

Interest in iron supplementation, namely with ferric carboxymaltose or ferric derisomaltose has increased significantly following emerging evidence that it improves QoL and alleviates the symptoms of HF. Numerous studies have therefore sought to clarify the additional value it brings to modern HF management [257,258,259,260,261,262,263,264,265,266,267,268,269,270,271,272,273,274,275,276,277,278,279,280,281,282,283,284,285,286,287,288,289].

Data from the Swedish Heart Failure Registry provided important real-world information, demonstrating how underdiagnosed iron deficiency (ID) is in HF patients and allowing the establishment of a link between the occurrence of ID in all HF phenotypes, not just HFrEF; the baseline prevalence was 55% (HFrEF 54%, HFmrEF 51%, HFpEF 61%) [279]. It is also worth noting that some ID may appear de novo, which highlights the need for thorough patient monitoring [279]. Additionally, in the Swedish registry, ID was associated with a higher risk of rehospitalization and composite endpoints, which confirms its prognostic value also outside RCTs [279].

Sharma et al. and Cabrera et al. reached a similar conclusion, stating that ID is a common phenomenon, occurring independently of anemia, especially in older adults [259,268]. Furthermore, Cabrera noticed that it is more common in patients with newly diagnosed HF and is associated with poorer QoL [259]. Another important aspect is the diagnostic value that ID brings to the risk stratification of patients with HF, namely, that it may be associated with an increased risk of hospitalization and death, as suggested by some researchers [259,263].

The assessments of the risk of hospitalization and mortality are of particular interest, as some of the primary analyses stated that iron supplementation reduces the risk of first HF hospitalization (HHF) [266], while secondary analyses indicated an overall reduction in the number of HF hospitalizations; however whether it reduced mortality as well remained inconclusive [257] until a meta-analysis by Graham et al. showed that IV iron supplementation actually reduces the risk of composite endpoints, including recurrent HHF + CVD (RR 0.75, 95% CI 0.61–0.93; *p* < 0.01) and first HHF or CVD (OR 0.72, 95% CI 0.53–0.99; *p* = 0.04), while showing no statistical significance for CV mortality (OR 0.86, 95% CI 0.70–1.05; *p* = 0.14) or all-cause mortality (OR 0.93, 95% CI 0.78–1.12; *p* = 0.47) alone [276]. It demonstrates that iron supplementation actually translates into a reduction in the number of HF hospitalizations, but it is currently impossible to establish a correlation between the described therapy and a reduction in mortality [276]. It is also worth noting that better benefits were achieved by patients with transferrin saturation (TSAT) below 20% (OR 0.67, 95% CI 0.49–0.92), while TSAT results above 20% essentially eliminated the significance of the therapy (OR 0.99, 95% CI 0.74–1.30) [276]. However, another analysis by Anker et al. showed no statistically significant differences between the individual populations: women vs. men; age < 69.4 vs. ≥69.4 years; HF etiology: ischemic vs. non-ischemic; eGFR: ≤60 vs. >60 mL/min/1.73 m^2^; hemoglobin: <11.8 vs. ≥11.8; ferritin: <35 vs. ≥35 μg/L; NYHA II vs. III/IV, and, very interestingly, also TSAT < 20% vs. ≥20% (RRR 0.75, CI 0.40–1.34), which is a counterargument to the previous analysis [270]. Therefore, this meta-analysis confirms no differences between the groups mentioned, and the beneficial effect of reducing the composite endpoint of HHF + CVD (RR 0.73, 95% CrI 0.48–0.99) is uniform for all the mentioned HF populations with ID [270].

Another aspect worth mentioning is the definition of ID, which seems to vary significantly depending on the interpretation. There are several definitions, including ferritin < 100 ng/mL or 100–299 ng/mL with TSAT < 20% and serum iron ≤ 13 μmol/L or low TSAT < 20% alone [271,288]. Authors draw attention to a crucial fact, based on the data from Papadopoulou et al.’s study, that the prevalence of ID ranges from 39% to 55%, depending on the definition used. Importantly, among patients identified as iron-deficient by any definition, only 51% met all three criteria, indicating that overlap between definitions occurs in roughly half of the ID population [271,288]. It follows that the ferritin-based definition may identify patients who do not have significant ID affecting cardiac function or clinical outcomes, and their simultaneous inclusion in a study based on this definition may lead to a dilutional effect when attempting to correlate ID with clinical outcomes [271]. This shows that in patients with ID and HF there is a need to correct the definition to better assess the actual clinically significant ID. This shows that in patients with ID and HF there is a need to correct the definition to better assess the actual clinically significant ID [271,288].

Finally, other notable observations regarding iron homeostasis in HF include an increase in organ iron stores, as detected by magnetic resonance imaging (MRI), following supplementation [261], as well as a significantly lower risk of hypophosphatemia after ferric carboxymaltose treatment in HF patients compared to other groups [265]. A summary of the most important findings regarding the therapies described in this subchapter is presented in Table S2.

## 5. Cardiomyopathies and Amyloidosis: Cutting-Edge Challenges and Discoveries

### 5.1. Cardiac Amyloidosis

Cardiac amyloidosis (CA) is an often underestimated disease that has recently been receiving increasing attention [290,291,292,293,294,295,296,297,298,299,300,301,302,303,304,305,306,307,308,309,310,311,312,313,314,315,316,317,318,319,320,321,322,323,324,325,326,327,328,329,330,331,332,333,334,335,336,337,338,339,340,341,342,343,344,345,346,347,348,349,350,351,352,353,354]. The risk of comorbid CA in HF patients was investigated by Berthelot et al., who observed a significantly higher risk of adverse events compared to patients with HF alone. Within 90 days after hospitalization for acute HF (AHF), the risk of rehospitalization in the HF + CA group was twice as high as in the HF-only group, and the risk of death was three times greater [296]. Despite the small sample size (N = 162), these results were highly statistically significant (*p* < 0.001) [296].

A substantial amount of data is available on the QoL of patients with CA. Surveys based on the Kansas City Cardiomyopathy Questionnaire (KCCQ) have shown that patients with amyloid transthyretin cardiomyopathy (ATTR-CM) experience a substantially worse QoL across multiple functional domains, even when compared with patients suffering from other cardiac diseases presenting with the same NYHA class severity [302]. This highlights the importance of early diagnosis and possible implementation of treatment, especially in patients with HFpEF [306].

Moreover, frailty has recently been identified as an additional prognostic factor in ATTR-CM, further compounding the negative impact on functional capacity and survival [337].

Comorbidities frequently accompany CA and strongly influence prognosis. Atrial fibrillation (AF) is particularly prevalent in ATTR-CM, where it represents both a consequence of atrial amyloid infiltration and a prognostic marker for increased mortality [335,354]. Light-chain pericardial amyloidosis has also been reported as an emerging phenotype alongside variant ATTR-CM, complicating both diagnosis and management [339]. Histological typing remains crucial to differentiate CA subtypes and guide treatment strategies, with ongoing refinements in tissue-based diagnostic approaches [338].

Genotype, race, and socioeconomic status also influence the presentation and outcomes of the disease. Black women carrying the amyloidogenic V122I transthyretin mutation show increased cardiovascular morbidity and mortality [340]. More broadly, socioeconomic disparities and racial differences significantly impact diagnosis rates and clinical outcomes in ATTR-CM [343,350]. These findings stress the need for equitable access to diagnostic resources and therapies.

Fortunately, increasing evidence supports the effectiveness of tafamidis in treating ATTR-CM. Twelve months of therapy has been associated not only with preservation of biventricular systolic function and reduction of left ventricle (LV) mass, but also with stabilization of myocardial structure and potential slowing of disease progression [301]. Long-term real-world data further confirm that tafamidis therapy is linked to sustained reductions in mortality and hospitalization rates, supporting its durable efficacy across heterogeneous patient populations [334,344]. At the same time, acoramidis, a novel transthyretin stabilizer, has demonstrated favorable effects on myocardial structure and function, suggesting that earlier initiation of therapy may translate into superior long-term outcomes [347]. Nevertheless, the clinical relevance of serial bone scintigraphy findings in treated patients remains uncertain, indicating the need for more robust imaging endpoints [348]. Collectively, these observations reflect the rapidly evolving therapeutic landscape of ATTR-CM [336]. However, the authors also note that early diagnosis and treatment implementation remain challenging; therefore, the use of biomarkers such as high-sensitivity cardiac troponin T (hs-cTnT), BNP, or eGFR could allow for better risk stratification and prediction of clinical outcomes, especially since patients treated with tafamidis show high adherence [301,310,318,321,322]. Recent data support expanding biomarker strategies beyond natriuretic peptides: high-sensitivity cardiac troponin I (hs-cTnI) has been shown to provide incremental prognostic information in wild-type ATTR-CM [333], while both hs-cTnT and NT-proBNP remain strong predictors of adverse outcomes across CA subtypes [352]. Serum albuminuria has also emerged as an independent predictor of disease progression [341]. Furthermore, capillary rarefaction within the myocardium has been reported as a structural hallmark of disease progression, with potential diagnostic and therapeutic implications [328]. Novel imaging studies have highlighted alterations in myocardial perfusion and the presence of microvascular obstruction as contributors to disease severity and adverse outcomes [331,345]. Expanded staging systems, such as the extension of the National Amyloidosis Centre staging system, now enable earlier detection and refined prognostic classification [346]. In parallel, the Mayo ATTR-CM score has been compared with other diagnostic scores, emphasizing the value of integrated biomarker-based risk stratification [330].

Finally, consensus documents now recommend a multidisciplinary approach to optimize care for patients with CA, integrating cardiology, hematology, neurology, and supportive care services [342]. Such holistic management is essential, given the disease’s complexity, its variable clinical phenotypes, and the growing therapeutic options available.

### 5.2. Takotsubo Cardiomyopathy

Takotsubo syndrome (TTS) is still a poorly understood disease, which is why some researchers focus their efforts on it [355,356,357,358,359,360,361,362,363,364,365,366,367,368,369,370]. Contemporary syntheses emphasize a multifactorial pathophysiology integrating catecholaminergic surges, the autonomic brain–heart axis, microvascular dysfunction, and immune activation [370]. Neuroimaging studies further support central involvement, demonstrating acute structural and functional brain changes that may contribute to susceptibility and clinical expression [368].

Studies on the pathophysiology of TTS indicate a significant correlation between coronary microcirculation dysfunction (CMD) and the development of this disease [357,363]. Specifically, the microcirculatory resistance index (IMR) was found to be elevated above 25 mmHg·s in 35% of the TTS patients studied. Additionally, researchers also observed a relationship between the disease and inflammatory mediators such as monocyte/macrophage activation, which may indicate the immunological nature of this disease [362,363]. Moreover, CMD was found to be more common in TTS than in ischemia and non-obstructive coronary artery disease (INOCA), and particularly severe changes in CMD could be observed in the apical phenotype of TTS compared to the mid-ventricular one [357]. Recent work on myocardial reverse remodeling underscores that functional recovery can be substantial yet incomplete in some patients, informing follow-up strategies and rehabilitation targets [367].

Regarding TTS pharmacotherapy, it remains problematic, but data from the SWEDEHEART registry provide some guidance. The study found that inotropic and diuretic agents were associated with increased 30-day mortality, respectively: (HR ≈ 9.9, *p* < 0.001), (HR ≈ 3.22, *p* = 0.001), while angiotensin-converting enzyme inhibitors (ACEIs) and statins reduced mortality, respectively: (HR ≈ 0.60, *p* = 0.025; HR ≈ 0.62, *p* = 0.040) [360]. It is also worth noting that in this study, beta-blockers, angiotensin receptor blockers (ARBs), and P2Y12 inhibitors did not show a significant correlation with mortality in the analysis [360]. These observations align with contemporary management statements that endorse individualized hemodynamic support, caution with catecholaminergic inotropes (especially when LVOTO is suspected), and selective use of ACEI/ARB and statins as part of comprehensive care [369].

An additional study suggests that in nondiabetic patients with TTS, stress-induced elevations in blood glucose levels result from catecholamine-induced insulin resistance and are associated with a poorer prognosis [355]. High blood glucose levels at the beginning of hospitalization correlated with parameters of an unfavorable disease course, which may be an indirect marker of increased catecholamine stress [355]. Parallel advances in risk stratification show that machine-learning models using clinical and ECG variables can improve prediction of in-hospital death and complications, supporting early triage and monitoring [365]. Moreover, longer-term data indicate that TTS is not uniformly benign and is associated with nontrivial cardiovascular mortality during follow-up, underscoring the need for structured surveillance after the acute episode [364].

### 5.3. Hypertrophic Cardiomyopathy

HCM is the most common primary cardiomyopathy, affecting approximately 1 in 500 people in the general population, making it an important area of cardiology research, represented by numerous studies [371,372,373,374,375,376,377,378,379,380,381,382,383,384,385,386,387,388,389,390,391,392,393,394,395,396,397,398,399,400,401,402,403,404,405,406,407,408,409,410,411,412,413,414,415,416,417,418,419,420,421,422,423,424,425,426,427]. Lifetime data analyses highlight distinct phases of progression, underscoring the heterogeneity of the disease course [415]. In addition, histopathological findings indicate that abnormalities of the mitral valve leaflet can contribute to LVOT obstruction and symptom severity [427].

Regarding HCM therapies, there are two paths that have so far been considered alternatives, although some new data show that therapy with the myosin inhibitor Mavacamten and alcohol septal ablation (ASA) should be considered complementary rather than alternative [372,373,378]. As indicated by Abood et al. and Achim et al., in some patients Mavacamten therapy may delay or eliminate the need for ASA, but the therapeutic decision is strongly dependent on factors such as the anatomy of the left ventricular outflow tract (LVOT), septal thickness and geometry, and valve abnormalities such as systolic anterior motion (SAM) or mitral regurgitation (MR) [372,373,384]. Real-world data confirm that a significant proportion of patients are candidates for Mavacamten, supporting its implementation outside clinical trials [399]. Importantly, beta-blockers remain relevant, even when combined with myosin inhibitors, to optimize hemodynamic outcomes [402]. Clinical trial evidence further consolidates the beneficial effects of Mavacamten in symptomatic obstructive patients [420].

In conclusion, both therapies, especially in a complementary approach, are correlated with improvement in the NYHA class, reduction of hemodynamic load, specifically a decrease in the LVOT gradient, with Mavacamten achieving great results (−49.4 mmHg (*p* < 0.001) at 4 weeks, −59.2 mmHg (*p* < 0.001) at 8 weeks, and −60.8 mmHg (*p* < 0.001) at 12 weeks); however, this is associated with the risk of a transient decrease in EF, hence the need for constant monitoring and titration of the Mavacamten dose [372,373,378]. Aficamten, a next-generation myosin inhibitor, has recently been shown to be effective in both obstructive and non-obstructive HCM, with improvements in gradients, cardiac structure, and PROs [411,412,413,421,425]. Moreover, studies on temporary withdrawal of standard-of-care medication during aficamten therapy have demonstrated feasibility and improved tolerability [414].

An additional critical consideration is the investigation of the impact of inflammation and fibrosis on the progression of HCM, as well as the potential strategies to mitigate these pathological processes [379,383].Research shows that in the HCM group, higher inflammatory markers, such as interleukin 2 (IL-2) and tumor necrosis factor α (TNF-α), can be observed, which were particularly associated with interstitial fibrosis, for IL-2 and TNF-α respectively: (OR: 1.49, 95% CI: 1.06–2.09, *p* = 0.021; OR:1.35, 95% CI: 1.01–1.80, *p* = 0.044) [383].As is well known, chronic inflammation is also associated with cardiac remodeling, which is particularly reflected in this patient group. This finding is particularly noteworthy in the context of the study by Filippatos et al., which demonstrated that the beneficial effects of finerenone on left ventricular remodeling are not attenuated in the presence of LVH [379]. Moreover, better treatment results were observed in hospitalized HF patients in the group with LVH than in the group without it; therefore, LVH may prove to be a good predictor of finerenone therapy, potentially also in the HCM group [379]. Although this study did not directly address the HCM, it points to an interesting research direction in which patients with HCM inflammatory/fibrotic profiles could benefit from the use of finerenone and possibly alleviate the inflammation-fibrosis-remodeling axis [379,383]. Other novel approaches include metabolic modulation, where ninerafaxstat showed safety and efficacy in non-obstructive HCM [410]. Furthermore, clonal haematopoiesis has been identified as a marker of increased cardiovascular risk [422], while radiomics of LGE reveals the prognostic value of myocardial scar heterogeneity [404].

Risk stratification in HCM is being refined by novel diagnostic strategies. Artificial intelligence-based ECG analysis improves recognition of disease in clinical practice [401]. Phenotype-based classification of patients undergoing myectomy enhances prediction of surgical outcomes [400]. Dedicated HCM heart failure risk models now enable more accurate prognosis [407]. Genetic testing remains of high diagnostic value, also in understudied populations [403].

Clinical heterogeneity is also expressed in special populations. Pregnancy in women with HCM carries unique long-term risks [398]. Patients with apical aneurysms or mid-ventricular obstruction are recognized as a high-risk phenotype with increased risk of arrhythmia and sudden cardiac death [423]. Elderly patients also benefit from invasive therapies, as survival analyses have confirmed positive outcomes after septal reduction even in patients ≥65 years [416]. In advanced cases, mechanical circulatory support and transplantation remain valid therapeutic options [408].

Overall, management of HCM is entering a new era of precision medicine. Guidelines highlight an integrated, multidisciplinary approach combining pharmacotherapies, interventions, genetics, and imaging [418]. In addition, perspectives on early-stage disease emphasize the importance of subclinical markers and QoL monitoring to ensure timely treatment initiation [405]. Finally, multimodality imaging retains a central role in diagnosing LVH and distinguishing HCM from other causes of hypertrophy [417].

## 6. Conclusions

Heart failure remains one of the leading causes of morbidity and mortality worldwide, despite notable therapeutic and diagnostic progress achieved in recent years. The syndrome is heterogeneous in both etiology and clinical course, which underscores the need for precise phenotyping and individualized management. The tripartite classification into HF with preserved (HFpEF), mildly reduced (HFmrEF), and reduced ejection fraction (HFrEF) has contributed to a more accurate understanding of the pathophysiology and treatment responses across the spectrum of left ventricular function.

Advances in pharmacotherapy, particularly the introduction of Glucagon-like peptide-1 receptor agonists (GLP-1a), sodium–glucose cotransporter-2 inhibitors (SGLT2i), and angiotensin receptor–neprilysin inhibitors (ARNIs), have substantially improved clinical outcomes and are now regarded as central components of guideline-directed medical therapy. Intravenous iron supplementation has emerged as an effective adjunctive strategy, primarily in reducing hospitalizations and improving QoL, although its impact on mortality requires further clarification. For patients with HFrEF, additional therapeutic modalities such as vericiguat and device-based interventions (e.g., baroreflex activation therapy) offer incremental benefits.

Beyond classical HF syndromes, the management of cardiomyopathies—including cardiac amyloidosis, Takotsubo syndrome, and hypertrophic cardiomyopathy—has been enriched by novel therapeutic options. Tafamidis and newer transthyretin stabilizers have redefined the prognosis of transthyretin amyloidosis, myosin inhibitors represent a breakthrough in hypertrophic cardiomyopathy, and structured surveillance has been recognized as essential in Takotsubo syndrome. These developments collectively demonstrate the importance of tailoring interventions to disease-specific mechanisms and patient subgroups.

Emerging tools, such as artificial intelligence, multimodality imaging, and novel biomarkers, hold significant potential for earlier diagnosis, more refined risk stratification, and optimized therapeutic decision-making. Furthermore, increased recognition of the roles of comorbidities, patient-reported outcomes, and socioeconomic determinants of health underscores the need for a comprehensive, multidisciplinary, and patient-centered approach to HF management.

In summary, while heart failure continues to impose a substantial global burden, the therapeutic landscape is undergoing rapid and transformative change. Continued integration of pharmacological, device-based, and supportive strategies, combined with advances in precision medicine and multidisciplinary care, is crucial for achieving further reductions in morbidity and mortality. Future research should focus on improving early detection, refining therapeutic personalization, and addressing the persistent gaps in outcomes across HF phenotypes.

## 7. Limitations

Although there has been significant growth in understanding the mechanisms and treatment of HF, the strength of evidence in different areas varies. There is strong clinical trial data for pharmacological treatment of HF, including medications such as GLP-1a, ARNIs, SGLT2i, and iron therapy. The evidence is not as strong, and in some cases limited, as in the areas of effective management of comorbidities, device therapy, rehabilitation, physical activity, and digital health management. Furthermore, some studies have reported conflicting results, small sample sizes, and short follow-up, limiting the evidence base. Several gaps remain in this regard. For example, better characterization of the pathophysiological mechanisms linking inflammation and fibrosis in HF phenotypes, identification of patient subgroups most likely to benefit from new therapies, and high-quality studies in understudied areas are needed. Filling these research gaps will be important for translating improved mechanistic understanding into improved clinical outcomes.

## Data Availability

No new data were created or analyzed in this study. Data sharing is not applicable to this article.

## Supplementary Materials

The following supporting information can be downloaded at: https://www.mdpi.com/article/10.3390/jcdd12120484/s1, Table S1: Recent Heart Failure Trials Organized by Phenotype: A Visual Summary of Key Findings; Table S2: Summary of Heart Failure Therapies, Trial Populations, and Key Outcomes.
