# Supplementary material for: Heart Failure in the Modern Era: A Narrative Overview of Recent Research from 2022–2025
Source: J Cardiovasc Dev Dis. 2025 Dec 10;12(12):484. doi: 10.3390/jcdd12120484 (PMC12733460; doi:10.3390/jcdd12120484)
Supplement: Supplementary file 1 [file jcdd-12-00484-s001.zip › jcdd-3954526-Supplementary.pdf]

Table S1: Recent Heart Failure Trials Organized by Phenotype: A Visual Summary of Key Findings

HF type	Heart failure with preserved ejection fraction	Heart failure with mildly reduced ejection fraction	Heart failure with reduced ejection fraction
Key findings	**Pathophysiology & Mechanistic Insights**<br>• Chronic low-grade inflammation is a central driver of HFpEF, supported by multiple recent biomarker-based studies (IL-6, hsCRP, adipokines).<br>• Epicardial adipose tissue (EAT) emerges as an active inflammatory organ, influencing microvascular dysfunction and myocardial stiffness through cytokine release.<br>• Packer's adipokine-centric model has gained traction, proposing adipose–inflammation interplay as a unifying mechanism in HFpEF progression.<br>• Microvascular inflammatory injury, detectable through PCATA on CCTA, is newly recognized as a surrogate of coronary inflammation relevant to HFpEF development.<br>• Oxidative stress–driven pathways further contribute to myocardial and vascular fibrosis, creating a mechanistic link between comorbidities and HFpEF remodeling.<br><br>**Comorbidity-Linked Discoveries**<br>• Atrial fibrillation is increasingly recognized as a mechanistic amplifier of HFpEF severity, not just a comorbidity.<br>• Catheter ablation in HFpEF + AF patients produces measurable physiological improvement (lower PCWP, higher peak VO_2, reduced NT-proBNP), identifying a potentially disease-modifying intervention.<br><br>**Diagnostics & Early Detection**<br>• Hyperlactatemia during exercise testing has emerged as a sensitive early marker of impaired oxidative metabolism in HFpEF.<br>• Simple ergometric tests (cycle protocols) are now validated as meaningful tools for detecting abnormal hemodynamic responses early in disease.<br>• PCATA imaging provides a novel, non-invasive correlate of coronary inflammatory activity relevant to HFpEF identification.<br>• Composite diagnostic algorithms and ML-derived scores (e.g., HF-DANAS) demonstrate superior risk stratification and diagnostic accuracy compared with natriuretic peptides alone.<br><br>**Phenotyping & Machine Learning**<br>• Machine-learning approaches have successfully identified distinct HFpEF phenogroups (e.g., AF-dominant, CKD/atherosclerosis-heavy, LVH-dominant), supporting precision-medicine strategies.<br>• ML models also highlight the clinical importance of previously under-recognized undiagnosed HFpEF, which carries a paradoxically worse long-term mortality despite fewer comorbidities.	**Therapeutic Advances**<br>• SGLT2 inhibitors are the first drug class demonstrating consistent benefit across the entire EF spectrum, including HFmrEF, with effects extending beyond HF symptom relief (QoL improvement, anti-inflammatory and antioxidant effects).<br>• Therapeutic benefits of dapagliflozin are robust across stressors, including the COVID-19 pandemic and across QRS durations, suggesting utility even in CRT non-responders.<br>• Finerenone shows significant reductions in HF hospitalizations and CV death with preserved safety in elderly HFmrEF populations, broadening MRA applicability.<br><br>**Beta-Blocker Evidence**<br>• New analyses suggest β-blockers are neutral to mildly beneficial, but their effectiveness declines progressively above EF ≈40–45%, indicating a potential EF-dependent therapeutic gradient.<br><br>**Sex-Specific Insights**<br>• Recent studies identify female sex as an independent modifier of prognosis in HFmrEF, associated with lower mortality but higher rehospitalization rates and greater cumulative years of life lost to HF.<br>• These findings suggest that HFmrEF management may require sex-specific intensification strategies.<br><br>**Understanding HFmrEF as a Phenotype**<br>• Updated analyses challenge the view of HFmrEF as a mere transition stage; emerging phenotypic data support partial biological distinctiveness, particularly in inflammation and remodeling profiles.<br>• Nonetheless, heterogeneity remains substantial, underscoring the need for phenotype-directed RCTs.	**Neuromodulation (BAT)**<br>• Baroreflex Activation Therapy delivers durable improvements in NYHA class, quality of life, 6-minute walk distance, NT-proBNP, and overall functional status.<br>• Evidence confirms BAT is safe but not mortality-reducing in advanced HFrEF, emphasizing the need to better identify responders.<br><br>**Vericiguat as Emerging "5th Pillar"**<br>• Recent registry data and trial analyses reinforce that vericiguat reduces CV death and HF hospitalization beyond the conventional four pillars, especially in recently decompensated HFrEF.<br>• Hemodynamic improvements (e.g., reduced PAWP) provide mechanistic support for clinical benefits.<br>• Ongoing VICTOR trial is expected to clarify positioning relative to existing GDMT.<br><br>**Novel Comorbidity Insights**<br>• Obstructive sleep apnea substantially increases early rehospitalization risk (≈23%), highlighting the importance of sleep management in HFrEF pathways.<br>• Depression is newly validated as an independent prognostic factor, associated with higher HF hospitalization/death and adverse biomarker/EF changes.<br>• Osteoporosis and vertebral fractures double the risk of CV death or worsening HF, revealing an underappreciated bone–heart interaction with prognostic relevance.

Table S2: Summary of Heart Failure Therapies, Trial Populations, and Key Outcomes

Therapeutic class	Sodium-glucose contransporter 2 inhibi- tors	Angiotensin receptor-neprilysin inhibi- tors	Iron supplementation and metabolism	GLP-1 receptor agonists
Studies and their findings	**1. EMPEROR-Preserved**<br>Population: HFpEF (EF ≥50%), wide comorbidity burden (AF, CKD, obesity).<br>Endpoint: Reduction in HF hospitalization + CV death; slowed eGFR decline; BMI-dependent weight loss at 52 weeks.<br>Key study: EMPEROR-Preserved (177,178,185,186,211,214,215).<br>**2. EMPEROR-Reduced / pooled EF-spectrum analyses**<br>Population: HFrEF + HFmrEF.<br>Endpoint: Consistent reduction in HF hospitalization and CV death across EF, renal function, AF, CKD, obesity, or MRA use.<br>Key study: EMPEROR analyses (193–200,202,203,211–215).<br>**3. Clemmer et al.**<br>Population: HFpEF with CKD and/or hypertension.<br>Endpoint: Benefit attributable partly to improvement in HFpEF comorbidities (CKD, HT).<br>Study: Clemmer et al. (177).<br>**4. Mechanistic studies (anti-inflammatory / anti-fibrotic / metabolic)**<br>Population: HFpEF cohorts.<br>Endpoint: Evidence that SGLT2i exert anti-inflammatory, anti-fibrotic, anti-congestive, metabolic and ferroptosis-related effects.<br>Studies: 170,175,180,183–185,190–192,212,220.<br>**5. Sotagliflozin – Pitt et al.**<br>Population: T2DM + recent worsening HF; therapy initiated before hospital discharge.<br>Endpoint: Reduction in HF events + CV death at 30 days (HR 0.49) and 90 days (HR 0.54); reduced all-cause mortali-ty (HR 0.39).<br>Study: Pitt et al. (209).<br>**6. Canagliflozin – eGFR-spectrum analysis**<br>Population: HF patients across full renal function range (low to preserved eGFR).<br>Endpoint: Reduction in CV deaths + total HF hospitalizations (event rate 0.72; CI 0.65–0.80).<br>Study: Canagliflozin analysis (217).<br>**7. EMPEROR-Preserved renal & metabolic deep-dive**<br>Population: HFpEF patients across BMI categories.<br>Endpoint: Slowed GFR decline (1.31–1.43 ml/min/1.73 m^2/year), BMI-dependent weight loss (−0.6 kg to −2.7 kg).<br>Study: EMPEROR-Preserved subanalyses (211,214).	**1. PARADIGM-HF**<br>Population: HFrEF.<br>Endpoint: Reduced CV death + HF hospitalization.<br>Study: PARADIGM-HF (221–242).<br>**2. PARAGON-HF**<br>Population: HFpEF (EF >45%).<br>Endpoint: Primary composite endpoint not met; possible benefit in women and EF 45–60%.<br>Study: PARAGON-HF (238).<br>**3. PARAGLIDE-HF**<br>Population: Recent worsening HF (WHF), EF >40%.<br>Endpoint: Greater NT-proBNP reduction vs valsartan; stron-gest effect in EF 40–60% & women; fewer renal events; higher symptomatic hypotension.<br>Study: PARAGLIDE-HF (237).<br>**4. Remodeling / imaging-based response**<br>Population: HFrEF + HFmrEF.<br>Endpoint: Reversal of adverse remodeling, improved LV dimensions; hemodynamic force (HDF) changes linked to treatment response.<br>Studies: 225,226,223.<br>**5. Mebazaa et al. – diuretic responsiveness**<br>Population: HFrEF.<br>Endpoint: Enhanced natriuresis & diuresis; improved respon-se to volume challenge; better loop-diuretic sensitivity.<br>Study: Mebazaa et al. (236).<br>**6. RDW / prognostic biomarkers in HFpEF subgroup**<br>Population: HFpEF from PARAGON-HF.<br>Endpoint: ARNI does not significantly change RDW but RDW correlates with adverse CV events.<br>Study: 221.<br>**7. Oncology-related cardiotoxicity**<br>Population: Breast-cancer patients with chemotherapy-induced cardiotoxicity.<br>Endpoint: Potential benefit of ARNI on LV remodeling and function (emerging evidence).<br>Study: 229.	**1. Swedish HF Registry**<br>Population: All HF phenotypes; ID prevalence: HFrEF 54%, HFmrEF 51%, HFpEF 61%.<br>Endpoint: ID associated with higher rehospitalization and worse composite outcomes; some ID develops de novo.<br>Study: Swedish HF Registry (265).<br>**2. Sharma et al. & Cabrera et al.**<br>Population: HF patients with and without anemia.<br>Endpoint: ID common in elderly; associated with poorer QoL; prognostic value independent of anemia.<br>Studies: 245,254.<br>**3. Graham et al. meta-analysis**<br>Population: HF + iron deficiency.<br>Endpoint: Reduced recurrent HF hospitalization + CV death (RR 0.75); reduced first HHF/CVD (OR 0.72); no mortality reduction.<br>Study: Graham et al. (262).<br>**4. Anker et al. meta-analysis**<br>Population: HF + ID across EF spectrum.<br>Endpoint: Uniform benefit across age, sex, etiology, renal function, Hb, ferritin, TSAT.<br>Study: Anker et al. (256).<br>**5. Papadopoulou et al. — diagnostic definition problems**<br>Population: HF with suspected ID.<br>Endpoint: ID prevalence varies 39–55% depending on defini-tion; only 51% overlap between definitions → need for re-standardization.<br>Study: Papadopoulou et al. (257,274).<br>**6. MRI iron-store studies**<br>Population: HF patients treated with IV iron.<br>Endpoint: Increased myocardial iron content post-supplementation.<br>Study: 247.<br>**7. Hypophosphatemia safety data**<br>Population: HF compared with non-HF groups receiving IV iron.<br>Endpoint: Lower risk of hypophosphatemia with FCM in HF vs other populations.<br>Study: 251.	**1. Haemodynamic monitoring studies**<br>Population: HF patients treated with semaglutide or tirzepati-de (mixed EF).<br>Endpoint: Reductions in systolic/diastolic/mean PAP; effects independent of GDMT/diuretic changes; correlation with weight loss.<br>Study: 180.<br>**2. Structural cardiac adaptations (CMR)**<br>Population: Obesity-related HFpEF.<br>Endpoint: Reduced LV mass and paracardiac adipose tissue; changes in chamber volumes.<br>Study: 91.<br>**3. Mechanistic myocardium studies**<br>Population: Human ventricular myocardium (HFpEF-like and HFrEF samples).<br>Endpoint: Reduced late INa, reduced SR Ca leak, improved Ca transients and contractility via GLP-1R-dependent pathways; effects comparable to CaMKII inhibition.<br>Study: 181.<br>**4. SELECT cardiovascular outcomes**<br>Population: Overweight/obesity + ASCVD, including pa-tients with HF (HFpEF and HFrEF).<br>Endpoint: Reduced MACE; reduced composite of CV death or HF events; consistent effects across EF spectrum; no HFrEF-specific safety concerns.<br>Study: 97.<br>**5. STEP-HFpEF / STEP-HFpEF DM**<br>Population: Obesity-related HFpEF with or without T2DM.<br>Endpoint: Improved symptoms, physical limitations, 6MWD, inflammatory markers, and body weight.<br>Studies: 93, 94.<br>**6. Meta-analyses of GLP-1 Ras**<br>Population: T2D and cardiometabolic-risk populations.<br>Endpoint: Reduced MACE, HF hospitalization, and kidney disease progression; effects unchanged by concurrent SGLT2 inhibitor use.<br>Study: 100.<br>**7. Modelling analyses (combination therapy)**<br>Population: High-risk cardiometabolic patients.<br>Endpoint: Projected gains in long-term event-free survival when GLP-1 RAs combined with SGLT2i and MRAs.<br>Study: 182.
